# Pharmacokinetic Modeling of [^11^C]GSK-189254, PET Tracer Targeting H_3_ Receptors, in Rat Brain

**DOI:** 10.1021/acs.molpharmaceut.1c00889

**Published:** 2022-02-16

**Authors:** Nafiseh Ghazanfari, Aren van Waarde, Janine Doorduin, Jürgen W. A. Sijbesma, Maria Kominia, Martin Koelewijn, Khaled Attia, Antoon T. M. Willemsen, Ton J. Visser, André Heeres, Rudi A. J. O. Dierckx, Erik F. J. de Vries, Philip H. Elsinga

**Affiliations:** † University Medical Center Groningen, Department of Nuclear Medicine and Molecular Imaging, University of Groningen, Groningen 9700 RB, The Netherlands; ‡ Symeres, Groningen 9747 AT, The Netherlands

**Keywords:** kinetic modeling, pharmacokinetics, receptor imaging, [^11^C]GSK189254, Histamine H_3_ receptor

## Abstract

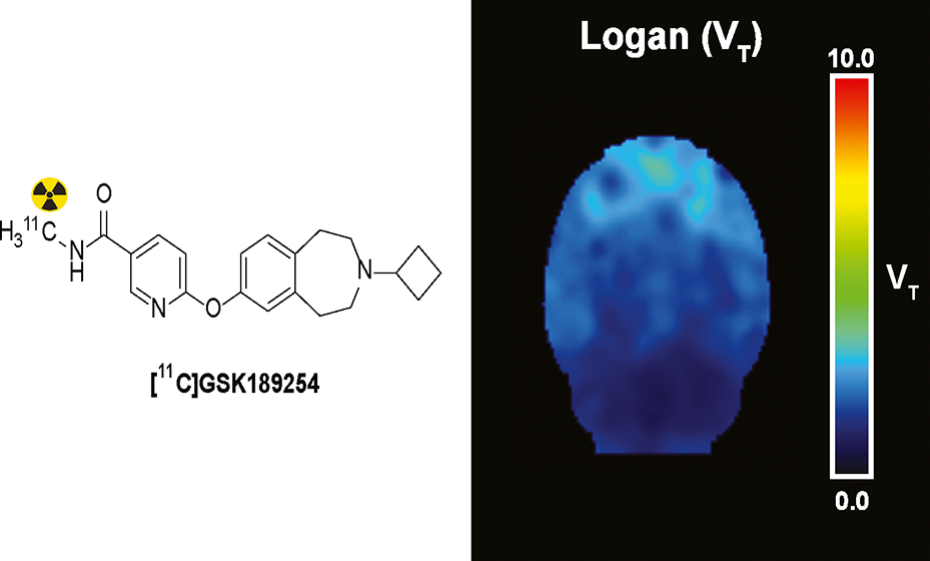

The histamine H_3_ receptor has been considered as a target for the treatment of various central nervous system diseases. Positron emission tomography (PET) studies with the radiolabeled potent and selective histamine H_3_ receptor antagonist [^11^C]GSK-189254 in rodents could be used to examine the mechanisms of action of novel therapeutic drugs or to assess changes of regional H_3_ receptor density in animal models of neurodegenerative disease. [^11^C]GSK-189254 was intravenously administered to healthy Wistar rats (*n* = 10), and a 60 min dynamic PET scan was carried out. Arterial blood samples were obtained during the scan to generate a metabolite-corrected plasma input function. PET data were analyzed using a one-tissue compartment model (1T2k), irreversible (2T3k) or reversible two-tissue compartment models (2T4k), graphical analysis (Logan and Patlak), reference tissue models (SRTM and SRTM2), and standard uptake values (SUVs). The Akaike information criterion and the standard error of the estimated parameters were used to select the most optimal quantification method. This study demonstrated that the 2T4k model with a fixed blood volume fraction and Logan graphical analysis can best describe the kinetics of [^11^C]GSK-189254 in the rat brain. SUV_40–60_ and the reference tissue-based measurements DVR(2T4k), BP_ND_(SRTM), and SUV ratio could also be used as a simplified method to estimate H_3_ receptor availability in case blood sampling is not feasible.

## Introduction

The histamine 3 (H_3_) receptor is one of the four members of the histamine receptor family. Histamine receptors are transmembrane G-protein-coupled (metabotropic) signaling proteins. A peculiar aspect of H_3_ receptors is the fact that they are constitutively active, which means that they display some signaling activities even in the absence of the endogenous agonist, histamine. H_3_ receptors have a global coordinating function^1,2^ and are highly expressed in the central nervous system (CNS), although they were first detected with low expression levels in peripheral organs.^3^ The H_3_ receptor can regulate the release of histamine in the CNS as a presynaptic autoreceptor. Histamine release in the mammalian brain shows a diurnal rhythm, with a low rate during sleep and a high rate during waking, active state.^4,5^ H_3_ receptors can also form heteroreceptors, which can modulate the release of other neurotransmitters, including acetylcholine,^6−8^ dopamine,^9,10^ noradrenaline,^11,12^ serotonin,^13,14^ and gamma-aminobutyric acid.^15^ Such neurotransmitters are essential for the normal operation of the brain.^5,16,17^

Evidence from animal studies and postmortem human brain analysis suggests that the H_3_ receptor is involved in several normal physiological processes and diseases or disorders of the brain, such as cognition,^5,18^ anxiety,^19^ sleep disorders,^16^ obesity,^20^ Alzheimer’s disease, Parkinson’s disease, schizophrenia, attention deficit hyperactive disorder, and epilepsy.^16,21−23^ Pharmacological studies have shown that blocking the constitutive activity of H_3_ receptors by the administration of an inverse agonist or antagonist enhances the release of histamine and may have therapeutic relevance.^4,24−27^ Therefore, the H_3_ receptor is considered as an interesting target in research concerning a wide spectrum of neurological and psychiatric diseases, and there is an increasing interest in the H_3_ receptor as a target for drug development^28^ and therapeutic intervention.^29^

Imaging techniques such as positron emission tomography (PET) could be a great tool in elucidating the role of the H_3_ receptor in normal physiology and disease. Various selective radiolabeled agonists and antagonists of H_3_ receptors have been developed as tracers for PET imaging.^30−44^ Some of these tracer candidates were not promising for clinical applications, but [^11^C]GSK-189254^1,45^ and [^11^C]AZ-12807110^46^ have already been used in clinical studies of the human brain.

As studies with [^11^C]GSK189254 for the quantitative PET imaging of H_3_ receptors in the rodent brain have not been reported yet, the aim of the present study is to determine the best model to describe the kinetics of histamine H_3_ receptor antagonist [^11^C]GSK-189254 and the feasibility of PET quantification without the need for rapid blood sampling in preclinical studies.

## Materials and Methods

### Tracer Synthesis

For the synthesis of [^11^C]GSK-189254, a screw-capped reaction vial (5 mL), equipped with a septum, was charged with a solution of GSK185071B (0.25 mg; Symeres, Groningen, The Netherlands) in anhydrous dimethyl sulfoxide (0.3 mL). A 2 M aqueous solution of sodium hydroxide (2 μL) was added to the precursor solution approximately 30 min prior to the addition of [^11^C]-methyl iodide. [^11^C]-methyl iodide was obtained from the reaction of [^11^C]-methane with iodine and bubbled into the reaction vial at room temperature until the radioactivity in the vial reached a maximum. The reaction mixture was heated at 80 °C for 4 min, after which a 0.1 M aqueous sodium bicarbonate solution (1.5 mL) was added. The reaction mixture was injected onto a semi-preparative high-performance liquid chromatography (HPLC) column (Gemini 5 μm C18 110 Å, 250 × 10 mm, 5 μm), which was eluted with 0.1 M ammonium acetate/acetonitrile (75:25 v/v) at a flow rate of 5 mL/min. The precursor GSK185071B was eluted at 8.5 min, and the product fraction containing [^11^C]GSK-189254 was eluted at approximately 10 min. The product fraction was diluted with water (80 mL) and passed through a Sep-Pak Plus C18 (light) cartridge, which was subsequently washed with water (2 × 8 mL). The pure product was obtained after elution of the cartridge with ethanol (0.8 mL) and aqueous phosphate-buffered saline (4.5 mL, pH 7.2). The eluted product was then filtered through a 0.22 μm Millex-LG sterilization filter and collected in a sterile vial.

### Animals

The animal experiments were performed in accordance with the regulations of the European Union (Directive 2010/63/EU) and the Dutch Experiments on Animals Act. The study protocol was approved by the Dutch National Committee on Animal Experiments (CCD: AVD1050020198648) and the Institutional Animal Care and Use Committee of the University of Groningen (IvD: 198648-01-004). Healthy male Wistar rats (*n* = 10 Hsd/Cpb:WU, aged 10 weeks, 366 ± 38 g) were purchased from Envigo (The Netherlands) and were housed in groups in a temperature (±21 °C)- and humidity (±40%)-controlled environment on a 12h light/dark cycle and with ad libitum access to food and water.

### PET Scan and Blood Data Acquisition

All PET scans were made between 10 and 12 a.m. because of the diurnal variation in histamine release.^47,48^ Rats were anesthetized with a mixture of isoflurane in oxygen (5% isoflurane for induction and 1–2.5% for maintenance). After anesthesia was induced, eye salve was applied to prevent dehydration of the cornea, and rats were placed on heating pads with electronic temperature controllers (M2M Imaging, Cleveland, OH, USA). Electronic temperature controllers were used to keep the body temperatures close to the normal value (set point of the heating pads was 38 °C). A cannula was placed in a femoral artery for arterial blood sampling, and a second cannula was placed in a tail vein for the administration of [^11^C]GSK-189254. Animals were placed in supine position with the head in the field of view of the PET camera (Focus 220 MicroPET, Siemens Healthcare, USA). A transmission scan using a ^57^Co point source to correct PET images for attenuation and scatter of radiation was followed by a dynamic emission scan, lasting 60 min. At the start of the emission scan, the tracer was injected over a period of 1 min using an infusion pump (1 mL/min). The administered dose of [^11^C]GSK-189254 was 50.5 ± 6.9 MBq. Blood oxygenation and the heart rate of rats were closely monitored during scans by using pulse oximeters (PulseSense, Nonin Medical, Plymouth, MN, USA), and the core temperature of the animals was recorded using rectal PTC thermometer probes and a data logging system (PicoTechnology, St. Neots, UK).

During the emission scan, a total of 15 blood samples with volumes between 0.1 and 0.15 mL were manually taken from the femoral artery at 10, 20, 30, 40, and 50 s and 1, 1.5, 2, 3, 5, 7.5, 10, 15, 30, and 60 min after the injection of [^11^C]GSK-189254 to generate an arterial input function for pharmacokinetic analysis. Heparinized saline was injected to compensate for the loss of blood by sampling. The total amount of blood withdrawn from each animal was kept below 2 mL (less than 10% of the total blood volume of a rat). One extra arterial blood sample was drawn at 2, 5, 10, 20, 30, 40, or 60 min to determine the fraction of the intact tracer in plasma, which was used to calculate a population-based metabolite curve. Plasma was separated from the whole blood by centrifugation (5 min at 30,000*g*). The total radioactivity in arterial plasma (25 μL) and whole blood (25 μL) was measured using a calibrated gamma counter (Wizard2480, PerkinElmer, USA). Results are presented as standardized uptake values (SUVs), which were calculated by dividing the measured activity concentration (kBq/mL) in each sample by the ratio of the injected dose (MBq) and the body weight (kg) of the animals.

### Plasma Parent Fraction

The fraction of intact [^11^C]GSK-189254 in arterial plasma samples was measured for the blood samples taken at 2, 5, 10, 20, 30, 40, and 60 min, as described above. After the separation of plasma from whole blood using a centrifuge for 5 min at 7500 rpm (Hettich Mikro 20, Hettich Zentrifugen, Germany), protein in the plasma sample was precipitated by adding an equal volume of acetonitrile and vortex-mixed for approximately 1 min. The protein-free supernatant was obtained by centrifuging the plasma–acetonitrile mixture for 3 min and filtration of the supernatant through a Millipore filter (Millex-HV 4-mm syringe filter, pore size 0.45 μm). The recovery of [^11^C]GSK-1989254-derived radioactivity from plasma after acetonitrile extraction was >95%. The filtrated supernatant was then injected onto a semi-preparative HPLC system using a Gemini C18 column (110 Å, 250 × 10 mm, 5 μm) with a mobile phase of 0.1 M ammonium acetate/acetonitrile (75:25 v/v) at a flow rate of 5.0 mL/min and UV detection (254 nm). Thirty second fractions of the eluate were collected and placed in a γ-counter (Wizard2480, PerkinElmer, USA) to measure the radioactivity. The percentage of intact [^11^C]GSK-189254 in plasma was calculated by comparing the area under the parent and metabolite peaks. The time-dependent metabolite fraction in plasma was calculated by fitting an exponential function to the data points obtained from the HPLC analysis. This population-based parent fraction curve was used to correct the plasma input function of individual rats for radioactive metabolites.

### PET Image Processing and Data Analysis

PET emission data were normalized and corrected for decay, scatter, random coincidences, and attenuation. The iterative reconstruction of data (OSEM2D, 4 iterations, and 16 subsets) resulted into temporal and spatial domain information composed of 21 frames (6 × 10, 4 × 30, 2 × 60, 1 × 120, 1 × 180, 4 × 30, and 3 × 600 s). All reconstructed PET images were automatically registered by rigid transformation to a tracer-specific brain template of [^11^C]GSK-189254, spatially aligned to a *T*_2_-weighted magnetic resonance imaging scan of the brain of a Wistar rat in Paxinos space.^49^ Image processing was performed using PMOD software (version 4.1; PMOD Technologies LLC, Zürich, Switzerland). Twelve volumes of interest (VOIs) were defined based on the rat brain atlas:^49^ parietal cortex, temporal cortex, occipital cortex, frontal cortex, striatum, amygdala, cerebellum, hippocampus, hypothalamus, brainstem, midbrain, and thalamus and a VOI covering the whole brain. Time-activity curves (TACs) in kBq/mL were generated for each predefined region and converted into time-dependent SUV curves.

### Pharmacokinetic Modeling

TACs of whole blood and metabolite-corrected plasma were determined and used as input functions for pharmacokinetic modeling using PMOD v4.1 software. Different compartment models were fitted to the TACs of the predefined brain regions, including a standard one-tissue compartment model (1T2k), irreversible two-tissue compartment model (2T3k), and reversible two-tissue compartment model (2T4k) using the cerebral blood volume fraction (*V*_B_) as either a fit parameter or a constant value fixed to 5% (*V*_B_ = 0.05).^50^ The individual kinetic rate constants *K*_1_, *k*_2_, *k*_3_, and *k*_4_ and the volume of distribution (*V*_T_), net influx rate (*K*_i_), and binding potential (BP_ND_) were estimated as outcome parameters using these models. In order to improve the quality of the fits in terms of the standard error (SE) of the estimated pharmacokinetic parameters, the *K*_1_/*k*_2_ ratio was incorporated in the 2T4k model as a constant parameter fixed to the value of the whole brain. Patlak and Logan graphical analysis was also applied, considering various starting times (t*) of 10, 20, and 30 min.

The optimal model was selected based on the Akaike information criterion (AIC). The reliability of estimated parameters was assessed based on the SE using a cutoff value of 25% and the coefficient of variance (COV). The accuracy of the macroparameters derived from the optimized models was determined through correlation analysis with the parameters from the corresponding cardinal compartment model, excluding all brain regions with an SE >25% for the estimated outcome parameter. We reported only descriptive statistics.

## Results

### Tracer Synthesis

Treatment of the labeling precursor GSK185071B with NaOH prior to the introduction of [^11^C]CH_3_I required a sufficient amount of time (i.e., 30 min) to ensure adequate deprotonation. Shorter treatment resulted in unsuccessful labeling. The radiotracer was obtained with a purity of 98.2 ± 1.0%, a molar activity of 19.1 ± 17.3 (GBq/μmol), and a decay-corrected radiochemical yield of 8.4 ± 6.5% with a total synthesis time of 42.8 ± 6.9 min.

### Blood, Plasma Tracer Kinetics, and Metabolism

**Figure 1:**
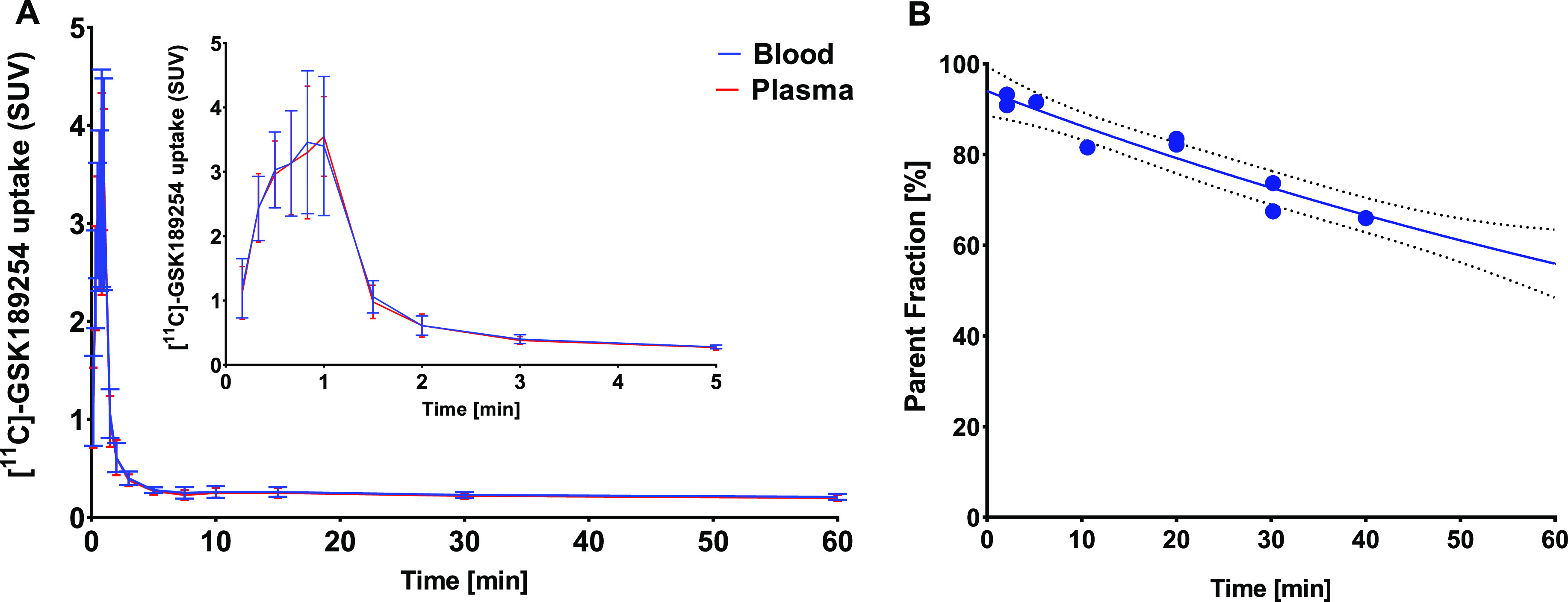
TACs of blood and plasma by manual sampling of arterial blood (A) and the inset represents the curve peak magnification, the population-based parent fraction in plasma (dashed lines show the 95% confidence intervals) (B); data are expressed as SUVs (mean ± SD).

Figure 1 shows the blood and plasma TACs and the percentage intact [^11^C]GSK-189254 during the 60 min dynamic PET scan. Using an infusion pump for injection (1 min infusion protocol) resulted in a peak concentration of [^11^C]GSK-189254 in whole blood and plasma at approximately 1 min after the start of tracer injection, followed by rapid clearance (Figure 1A). The population-based parent curve in plasma was well fitted by a monoexponential function with a half-life of 52.7 min (*R*^2^ = 0.93); 56.5% of plasma radioactivity still represented intact parent at 62 min post tracer injection (Figure 1B).

### [^11^C]GSK-189254 Uptake in Brain Regions

**Figure 2:**
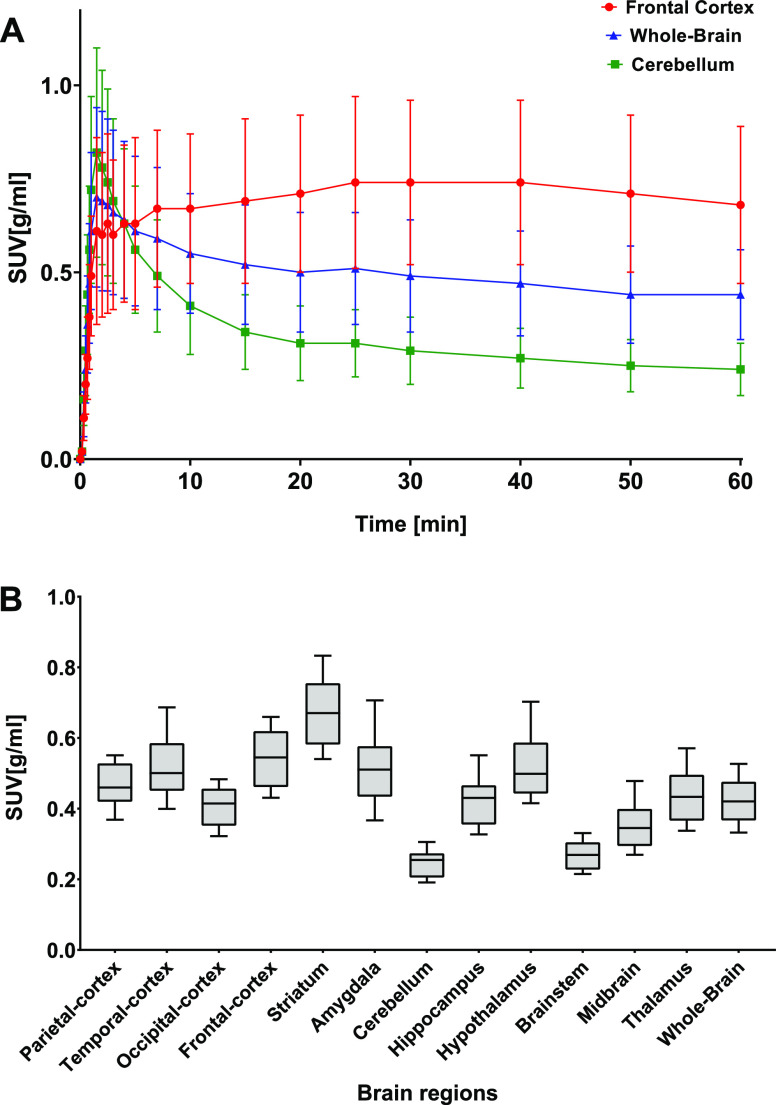
(A) [^11^C]GSK-189254 TACs of the striatum (high H_3_ receptor expression), cerebellum (low H_3_ receptor expression), and the whole-brain [^11^C]GSK-189254 quantification in brain regions including the striatum (high H_3_ receptor expression), cerebellum (low H_3_ receptor expression), and the whole brain using SUV and (B) regional SUVs for the last 20 min of the [^11^C]GSK-189254 scan (mean ± SD).

The kinetics of [^11^C]GSK-189254 in brain regions represented as TACs of the whole brain, striatum, and cerebellum is shown in Figure 2A. High uptake of [^11^C]GSK-189254 was observed in the striatum, followed by hypothalamus, amygdala, temporal, and frontal cortex. Cerebellum and midbrain, regions with low receptor expression, showed the lowest tracer uptake (Figure 2B).

Maximum tracer accumulation in the cerebellum and striatum was reached at 3.8 ± 4.9 and 25.1 ± 11.5 min after tracer injection, respectively. Significant washout was subsequently observed in the cerebellum, but not in the striatum, and tracer retention in both regions remained nearly constant for the last 30 min of the scan.

### Pharmacokinetic Modeling

#### Compartment Model

**Figure 3:**
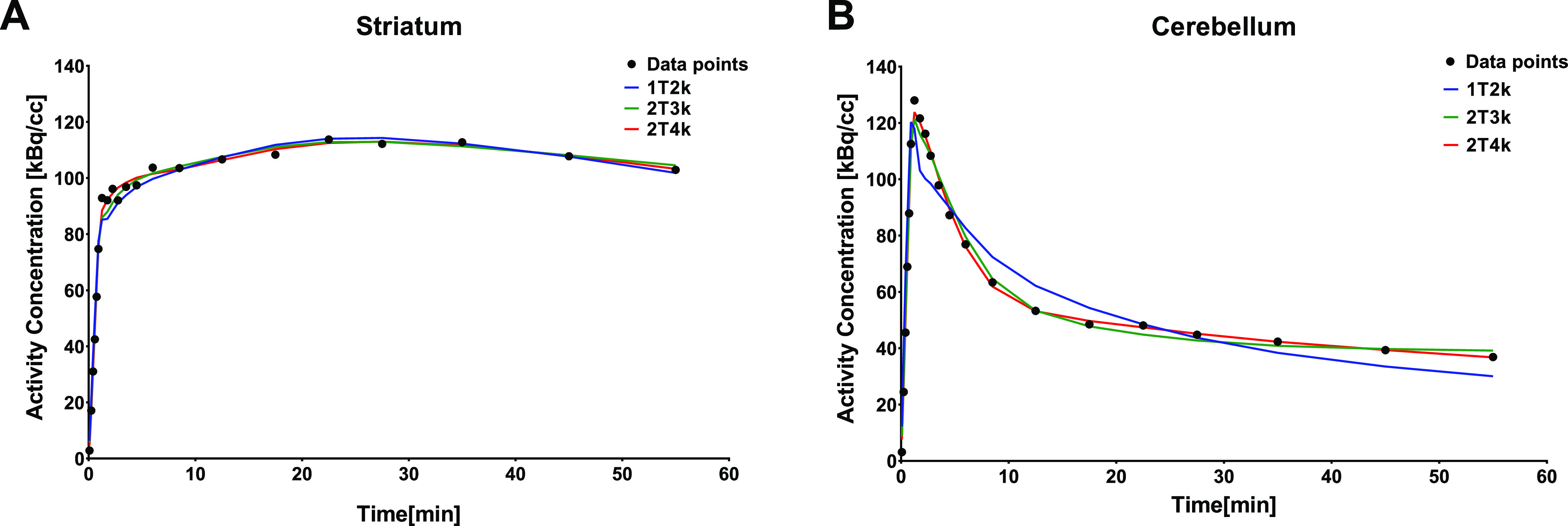
Representative fits of 1T2k (blue), 2T3k (green), and 2T4k (red) models to the actual data points (black) of the striatum (A) and cerebellum (B) TACs.

Visual assessment of the TACs revealed that the 1T2k and 2T3k models fitted the data less well than the 2T4k model (Figure 3). These differences were more pronounced in regions with low tracer binding (e.g., cerebellum) than in regions with high tracer binding (e.g., striatum).

**Figure 4:**
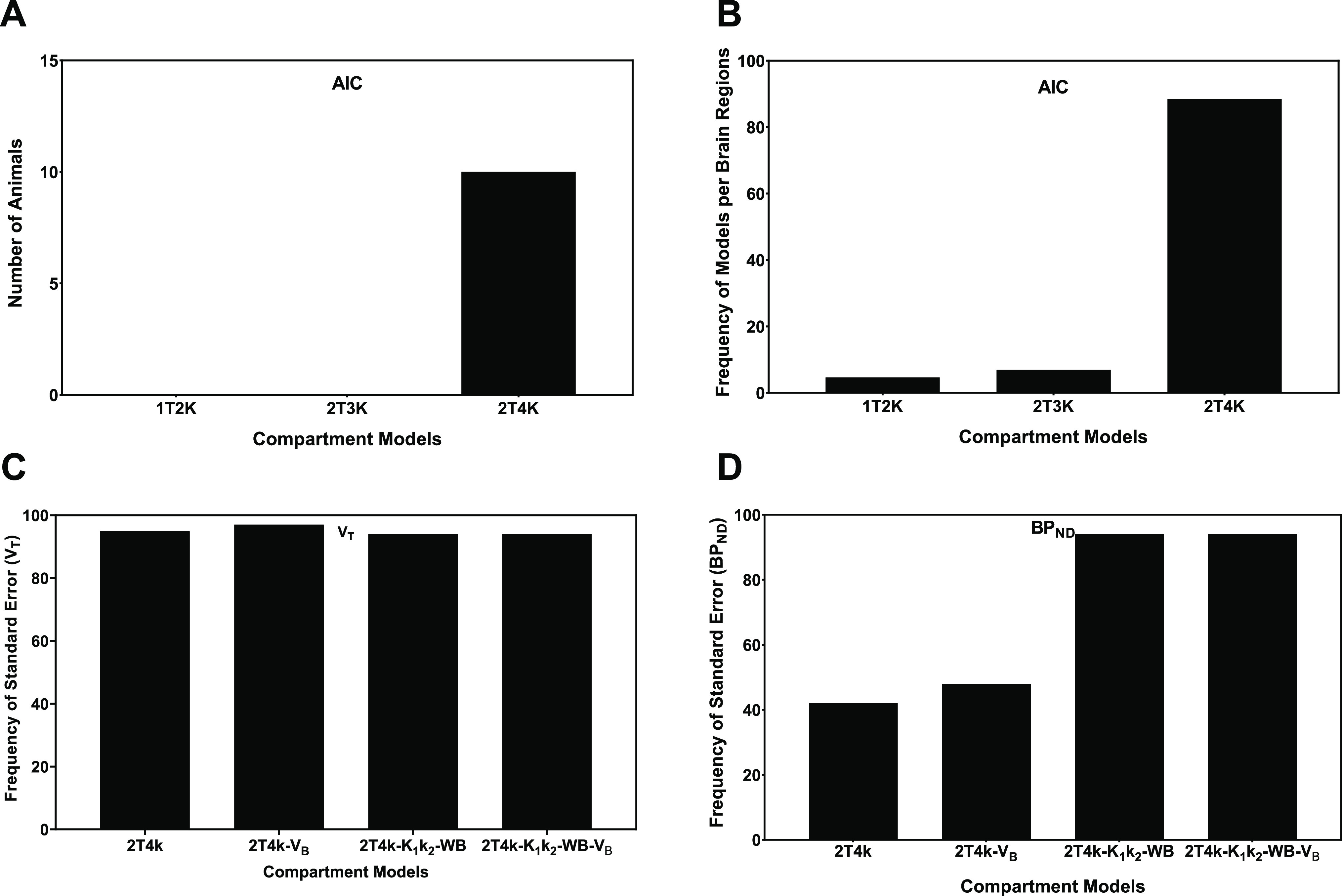
AIC-based model preference. (A) Number of animals and (B) number of brain regions with the lowest AIC value for a particular compartment model, as compared to the other models. The percentage of brain regions with SEs in the *V*_T_ (C) and BP_ND_ (D) <25%. *V*_T_ and BP_ND_ were estimated from the 2T4k model with or without fixing *V*_B_ to 0.05 and the *K*_1_/*k*_2_ ratio to the values of the whole brain.

Next, the AIC values were used as criteria to determine the quality of the fit of the one- or two-tissue compartment models (Supporting Information, Table 1). In all animals and almost all brain regions, the 2T4k model fit resulted in a lower AIC value than the other compartment models (Figure 4).

Thus, based on the AIC criteria (Figure 4A,B), the 2T4k model was selected as the model of choice for the quantification of [^11^C]GSK-189254. However, the SEs of the estimated fit parameters, specifically those of BP_ND_, were high. In fact, 58% of the estimated BP_ND_ values were imprecise (SE >25%).

Since the precision of the estimated BP_ND_ values was insufficient, we tried to improve the quality of the fits of the 2T4k model by applying constraints. This will reduce the number of variables that need to be estimated and thus should improve the robustness of the estimations.

It was assumed that the influx and efflux rate of [^11^C]GSK-189254 to/from the brain via passive diffusion is similar in all brain regions and consequently *K*_1_/*k*_2_ could be considered constant and thus could be fixed for the entire brain. The *K*_1_/*k*_2_ ratio in the whole brain was estimated in each scan and used as a fixed parameter in subsequent regional model fits (2T4k-*K*_1_*k*_2_). Furthermore, *V*_B_ was either fitted or fixed at 0.05 (2T4k-*V*_B_ and 2T4k-*K*_1_*k*_2_-*V*_B_) as approximately 5% of the brain consists of the cerebral blood.^50^ Fixation of *V*_B_ to 0.05 slightly improved the robustness of the fit (2T4k-*V*_B_) as the percentage of estimated *V*_T_ and BP_ND_ values with a SE >25% slightly decreased to 3 and 52%, respectively. Fixing the *K*_1_/*k*_2_ ratio to the values derived from the whole brain (2T4k-*K*_1_*k*_2_) strongly enhanced the robustness of the BP_ND_ estimations. The percentage of estimated BP_ND_ values with a SE >25% was reduced from 58% (2T4k) to 6% (2T4k-*K*_1_*k*_2_). In contrast, the percentage of *V*_T_ estimates with a SE >25% slightly increased from 5 to 6%. Fixation of both *V*_B_ and the *K*_1_/*k*_2_ ratio did not further improve the robustness of the fit as the percentage of *V*_T_ and BP_ND_ estimations with a SE >25% remained almost the same (6 and 8%, respectively). Taken together, these results suggest that most robust *V*_T_ estimates can be obtained with the 2T4k-*V*_B_ model, whereas most robust estimations of BP_ND_ can be obtained with the 2T4k-*K*_1_*k*_2_ model.

**Figure 5:**
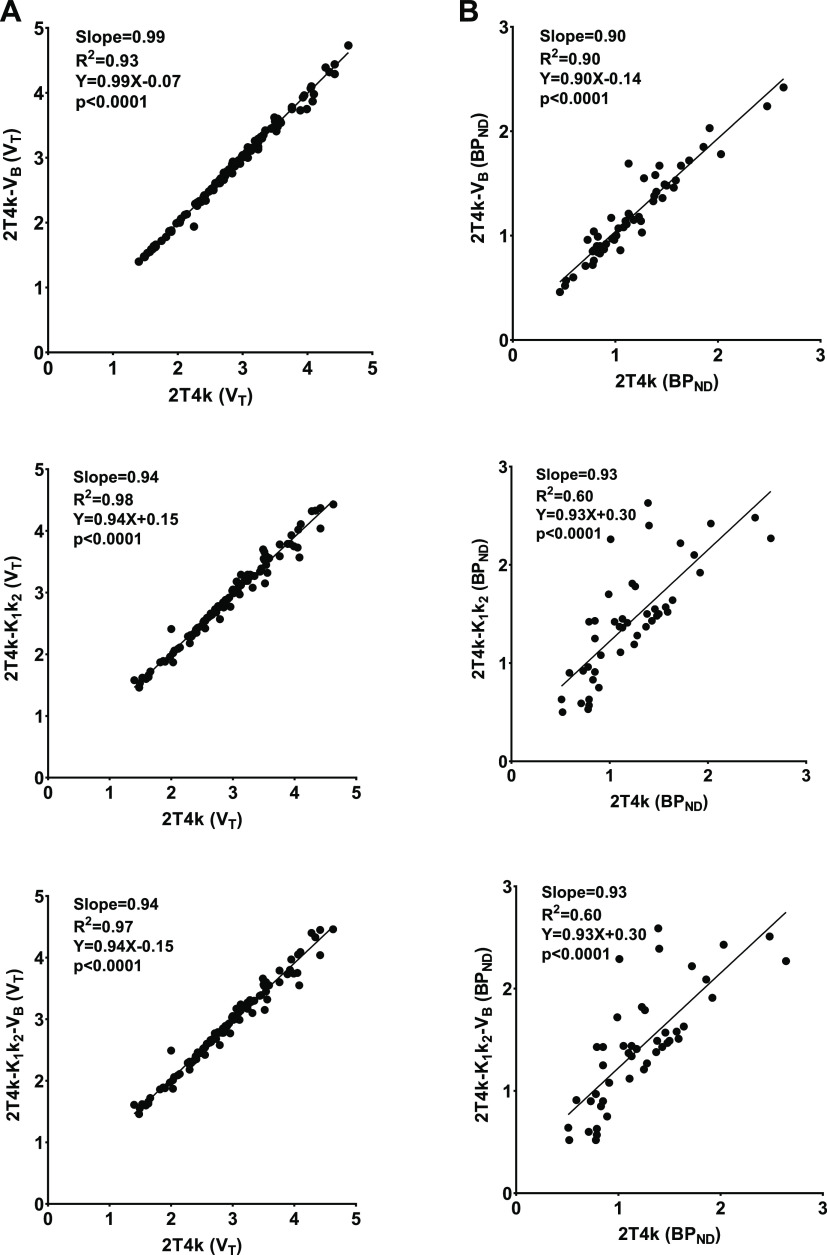
Correlation of *V*_T_ (A, left) and BP_ND_ (B, right) values derived from 2T4k and 2T4k-*V*_B_, 2T4k-*K*_1_*k*_2_, and 2T4k-*K*_1_*k*_2_-*V*_B_.

The correlation between optimized models and the cardinal compartment model was determined to find whether the optimization did influence the quantification accuracy and precision, with the *R*^2^ and slope of the correlation being related to the precision and accuracy, respectively. In general, the *V*_T_ values assessed with the models with fixed parameters (2T4k-*V*_B_, 2T4k-*K*_1_*k*_2_, and 2T4k-*K*_1_*k*_2_-*V*_B_) all showed excellent correlations with *V*_T_ values derived from the cardinal 2T4k model, with *R*^2^ values of 0.93–0.98, slopes of 0.94–0.99, and offsets of 0.07–0.15, as shown in Figure 5.

Based on the correlations, there was no a clear preference for any of the models with fixed parameters for estimating the *V*_T_ values. However, the COV of the *V*_T_ values estimated with the 2T4k-*V*_B_ model (11.8%; range 8–17%) was substantially lower than the COV for the 2T4k (23.6%; range 9–86%), 2T4k-*K*_1_*k*_2_ (16.7%; range 8–77%), and 2T4k-*K*_1_*k*_2_-*V*_B_ models (36.4%; range 8–88%). The highest COV was observed in the cerebellum and brainstem, areas with low H_3_ receptor density. When comparing the estimated BP_ND_ values, the correlation of 2T4k-*V*_B_ modeling estimates with the cardinal 2T4k estimates was clearly much better (*R*^2^ = 0.90) than the correlations for the models with a fixed *K*_1_/*k*_2_ ratio (*R*^2^ = 0.60). The values of the output parameters assessed with the most suitable models are provided in Table 1. The estimated parameters of *V*_T_ and BP_ND_ showed highest values in the striatum, cortex, thalamus, hippocampus, hypothalamus, and amygdala. Taking into account the AIC, the SEs in the estimated BP_ND_ values, the correlation with the cardinal 2T4k model, and the COV, it can be concluded that BP_ND_ could not be estimated as a reliable outcome parameter with any of the compartment models. In contrast, *V*_T_ values estimated with the 2T4K-*V*_B_ model could serve as a reliable outcome parameter.

**Table 1:** Estimated Values for BP_ND_ and *V*_T_ Derived from the Selected Optimized Models and the SUVs for the Last 20 min of the Scan (SUV_40–60_)^a^

	2T4k (*V*_T_)	2T4k-*V*_B_ (*V*_T_)	2T4k-*K*_1_*k*_2_ (BP_ND_)	Logan (*t = 30) (*V*_T_)	SUV_40–60_
brain regions	mean ± SD	mean ± SD	mean ± SD	mean ± SD	mean ± SD
parietal-cortex	3.00 ± 0.28	2.77 ± 0.29	1.81 ± 0.39	2.93 ± 0.27	0.47 ± 0.06
temporal-cortex	3.40 ± 0.40	3.39 ± 0.38	2.21 ± 0.64	3.22 ± 0.38	0.52 ± 0.09
occipital-cortex	2.64 ± 0.38	2.63 ± 0.35	1.43 ± 0.34	2.49 ± 0.28	0.41 ± 0.06
frontal-cortex	3.59 ± 0.42	3.60 ± 0.39	2.32 ± 0.49	3.40 ± 0.35	0.54 ± 0.08
striatum	4.12 ± 0.49	4.15 ± 0.49	3.24 ± 0.75	4.28 ± 0.47	0.67 ± 0.10
amygdala	3.45 ± 0.55	3.44 ± 0.58	2.23 ± 0.68	3.20 ± 0.45	0.51 ± 0.10
cerebellum	1.67 ± 0.25	1.62 ± 0.17	0.92 ± 1.03	1.56 ± 0.15	0.24 ± 0.04
hippocampus	2.78 ± 1.48	2.33 ± 0.23	1.48 ± 0.43	2.57 ± 0.33	0.42 ± 0.07
hypothalamus	4.55 ± 3.87	3.42 ± 0.50	2.12 ± 0.58	3.20 ± 0.28	0.52 ± 0.09
brainstem	1.72 ± 0.18	1.71 ± 0.17	0.85 ± 0.27	1.68 ± 0.18	0.27 ± 0.04
midbrain	2.23 ± 0.21	2.21 ± 0.20	1.08 ± 0.42	2.13 ± 0.19	0.35 ± 0.07
thalamus	3.26 ± 1.60	2.85 ± 0.43	1.58 ± 0.50	2.68 ± 0.32	0.44 ± 0.08
Whole brain	2.73 ± 0.28	2.71 ± 0.26	1.58 ± 0.39	2.62 ± 0.24	0.42 ± 0.06
COV%	23.6% [9–86%]	11.8% [8–17%]	34.2% [21–112%]	9.3% [7–11%]	16.1% [13–20%]

a Data are presented as mean ± SD.

#### Graphical Analysis

**Figure 6:**
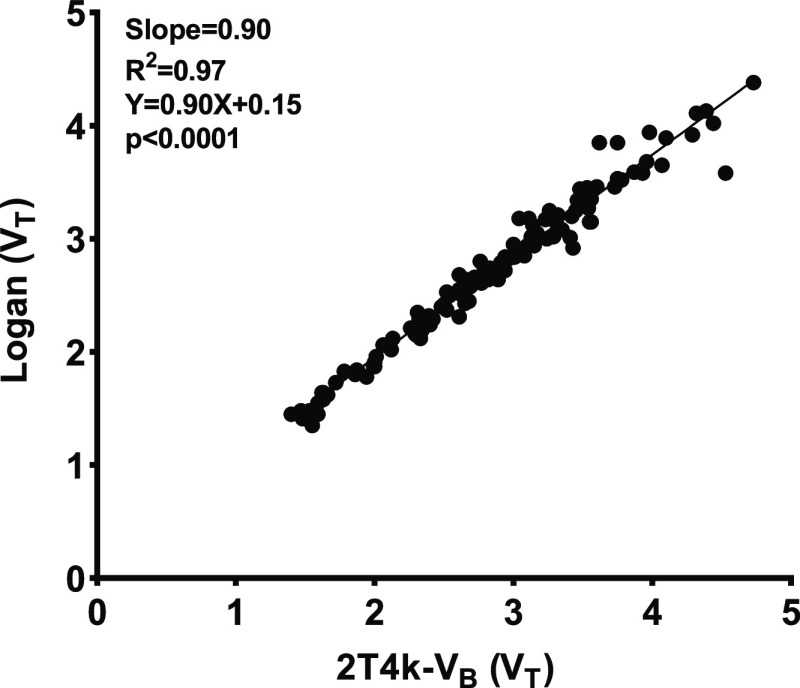
Regression analysis of the distribution volume (*V*_T_) of [^11^C]GSK-189254 in individual regions of the rat brain, determined by 2T4k-*V*_B_ compartment modeling and Logan graphical analysis.

Graphical methods such as Logan and Patlak graphical analysis often provide more robust modeling parameters than compartment models. Only Logan graphical analysis could fit the [^11^C]GSK-189254 kinetics in the brain (indicating reversibility of tracer binding). The obtained *V*_T_ values from 2T4k-*V*_B_ showed an excellent correlation with *V*_T_ derived from Logan graphical analysis when a starting time of 30 min was applied (*R*^2^ = 0.97, slope = 0.90, *p* < 0.0001, Figure 6). Similarly, high correlations were observed if the starting time was set to 10 or 20 min (*R*^2^ = 0.96, slope = 0.88, *p* < 0.0001 and *R*^2^ = 0.97, slope = 0.88, *p* < 0.0001, respectively).

The use of starting times of 10, 20, and 30 min did not affect *R*^2^ considerably and estimated values for *V*_T_ remained approximately the same. Although all starting times (*t) showed comparable correlations with the compartment model, we have chosen Logan graphical analysis with a starting time of 30 min based on the slightly higher slope of the correlation, suggesting slightly less bias. Thus, both the *V*_T_ from Logan analysis and the *V*_T_ from the optimal compartment model fit appeared useful for the quantification of [^11^C]-GSK-189254 binding.

#### Reference Tissue Models

Unlike compartment models and Logan graphical analysis, reference tissue models do not require an arterial input function. Based on the low *V*_T_ and BP_ND_ values of [^11^C]GSK-189254 in the cerebellum and brainstem, corresponding with low H_3_ expression levels as described in the literature,^51^ we evaluated these two brain regions as reference tissues in SRTM and SRTM2 model fits to explore the possibility of using reference tissue models rather than full kinetic modeling for data analysis. The estimated BP_ND_ values by SRTM and SRTM2 fits are presented in Table 2.

**Table 2:** SUV Ratio (SUVr) and BP_ND_ Values Obtained with SRTM and SRTM2 Using the Cerebellum and Brainstem as Reference Regions

	cerebellum as a reference region			brainstem as a reference region		
	SRTM	SRTM2	SUVr	SRTM	SRTM2	SUVr
brain regions	mean ± SD	mean ± SD	mean ± SD	mean ± SD	mean ± SD	mean ± SD
parietal-cortex	0.87 ± 0.09	0.87 ± 0.09	1.93 ± 0.12	0.67 ± 0.09	0.67 ± 0.09	1.75 ± 0.10
temporal-cortex	1.06 ± 0.13	1.00 ± 0.12	2.13 ± 0.13	0.85 ± 0.15	0.79 ± 0.15	1.94 ± 0.15
occipital-cortex	0.62 ± 0.05	0.61 ± 0.04	1.67 ± 0.07	0.45 ± 0.08	0.43 ± 0.07	1.52 ± 0.08
frontal-cortex	1.16 ± 0.10	1.14 ± 0.10	2.23 ± 0.12	0.94 ± 0.13	0.92 ± 0.13	2.02 ± 0.14
striatum	1.66 ± 0.11	1.66 ± 0.11	2.77 ± 0.13	1.40 ± 0.14	1.40 ± 0.14	2.51 ± 0.14
amygdala	1.02 ± 0.15	0.94 ± 0.13	2.10 ± 0.14	0.83 ± 0.17	0.73 ± 0.14	1.91 ± 0.15
hippocampus	0.66 ± 0.05	0.65 ± 0.05	1.73 ± 0.07	0.48 ± 0.08	0.47 ± 0.09	0.91 ± 0.05
hypothalamus	1.08 ± 0.08	1.05 ± 0.09	2.13 ± 0.11	0.86 ± 0.08	0.82 ± 0.07	1.57 ± 0.10
midbrain	0.44 ± 0.08	0.44 ± 0.08	1.10 ± 0.05	0.28 ± 0.06	0.28 ± 0.06	1.30 ± 0.09
thalamus	0.74 ± 0.07	0.74 ± 0.08	1.43 ± 0.10	0.54 ± 0.07	0.54 ± 0.06	1.62 ± 0.08
whole brain	0.68 ± 0.05	0.64 ± 0.05	1.78 ± 0.08	0.51 ± 0.07	0.46 ± 0.06	1.57 ± 0.08
COV%	8.5% [6–19%]	8.5% [6–19%]	5.2% [3–7%]	12.8% [9–22%]	12.7% [8–22%]	6.1% [4–8%]

The reference tissue-based models SRTM and SRTM2 with the cerebellum and brainstem as the reference tissue demonstrated poor correlations with BP_ND_ derived from the 2T4k-*V*_B_ compartmental model using a plasma input function, with *R*^2^ values ≤0.17 for all correlations (Supporting Information, Figure 1). However, BP_ND_ values estimated with SRTM or SRTM2 were highly correlated with the distribution volume ratio (DVR) values derived from the 2T4k-*V*_B_ compartment model using either the cerebellum or brainstem as reference regions (*R*^2^ = 0.89–0.91, *p* < 0.0001, Supporting Information, Figure 4).

#### Standardized Uptake Values

**Figure 7:**
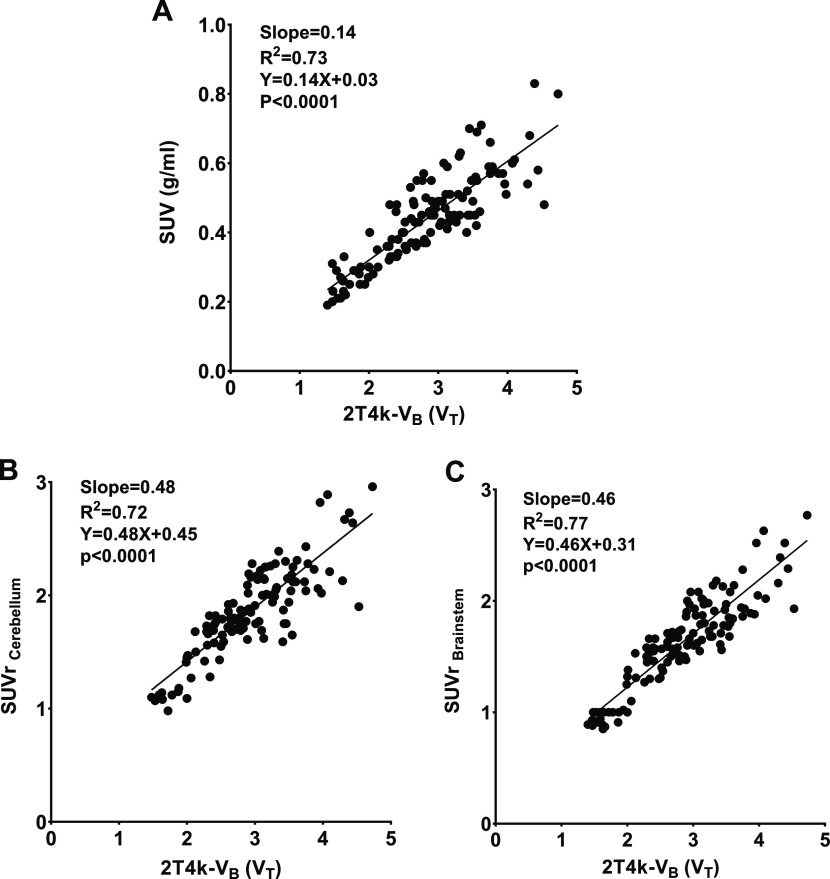
Linear Pearson correlation of (A) regional SUVs between 40 and 60 min after the injection of [^11^C]GSK-189254 (SUV_40–60_) for different brain regions and SUVr using the (B) cerebellum and (C) brainstem as the reference tissue, with the volume of distribution (*V*_T_) derived from the optimal compartment model (2T4k-*V*_B_).

We investigated whether SUV could be used as an easily assessable and robust parameter for assessing [^11^C]GSK-189254 binding. The SUVs derived from the last 20 min of the scans, when tracer kinetics was relatively stable, were calculated. The SUV_40–60_ ranged from 0.27 to 0.67 (COV: 16.1%, range 13–20%, Table 1) for various brain regions. The striatum, frontal cortex, temporal cortex, hypothalamus, and amygdala showed the highest uptake. Correlations between SUVs and *V*_T_ from the 2T4k-*V*_B_ model are shown in Figure 7. The SUVs of [^11^C]GSK-189254 showed a good positive correlation (*R*^2^ = 0.73) with the *V*_T_ derived from the optimal compartment model. Interestingly, the tissue-to-reference tissue (region) ratio considering the cerebellum and brainstem as the reference regions also provided a good correlation with *V*_T_ from the 2T4k compartmental model [SUVr (cerebellum): *R*^2^ = 0.72, *p* < 0.0001 and SUVr (brainstem): *R*^2^ = 0.77, *p* < 0.0001]. The averages of COV for the SUVr (cerebellum) and SUVr (brainstem) were 5.2% (range: 2–7%) and 6.1% (range: 4–8%), respectively.

Moreover, linear regression of the DVR of [^11^C]GSK-189254 in individual regions of the rat brain showed good correlations with the tissue-to-reference tissue ratio (SUVr) using either the cerebellum (*R*^2^ = 0.89, *p* < 0.0001) or brainstem (*R*^2^ = 0.89, *p* < 0.0001) as the reference region (Supporting Information, Figure 5).

## Discussion

We report here the first evaluation of [^11^C]GSK-189254 as a tool to assess H_3_ receptor density in the rat brain. Our findings indicate that the binding of [^11^C]GSK-189254 can be quantified well using *V*_T_ estimated with the 2T4k-*V*_B_ compartment model or Logan graphical analysis with a t* of 30 min. These models were selected based on the demonstrated good visual fits, low AIC values, and small SE and COV. In case arterial blood sampling is not feasible, SUV_40–60_ or SUVr with the cerebellum or brainstem as the reference tissue could be used as an acceptable alternative.

The estimated outcome (*V*_T_) value and regional uptake (SUV) of [^11^C]GSK-189254 from our study were in accordance with the known distribution of the H_3_ receptor in the rat brain, as determined by autoradiography.^51^ In particular, high levels of H_3_ receptors were observed in the striatum, frontal cortex, and olfactory bulbs. The olfactory bulbs were not included in the rat brain atlas used for the quantification of tracer uptake, but visual assessment of the PET images confirmed that tracer uptake was high in this brain region as well (Supporting Information, Figure 3).

The two-tissue compartment model (2T4k) was the most promising model for the quantification of [^11^C]GSK-189254 binding in the rat brain. However, high levels of uncertainty in the estimated fit parameters, especially BP_ND_ values (60% of the brain regions with an SE >25%), were observed. The difficulty in acquiring reliable BP_ND_ values could originate from the fact that estimating BP_ND_ can be challenging if the *k*_3_ and/or *k*_4_ values are small. Due to the noisy PET data, the convolution of noise level to the small measured values of *k*_4_ and *k*_3_ would result in large uncertainty in these parameters and consequently a poor BP_ND_ estimation, which is reflected by a large SE. Our observations were in agreement with findings from a human study.^1^ In that study, constraining both the *K*_1_/*k*_2_ ratio and *k*_4_ globally across all defined brain regions for each individual subject was required to enable the quantification of [^11^C]GSK-189254 in the human brain. Applying these major restrictions limits the degrees of freedom and thus the power of the model fit by converting variable parameters into constant values. Fixing parameters will generally increase the precision (lower SE) since there are less variables to estimate, but it may negatively affect the accuracy by introducing bias, causing the estimated value of the parameter to deviate from the true value. In our study in rats, we found that fixing *K*_1_/*k*_2_ to the *K*_1_/*k*_2_ ratio of the whole brain did not have a considerable influence on *V*_T_ estimates, while the levels of uncertainty in BP_ND_ estimates remained high. Fixation of *V*_B_ also did not substantially improve the robustness of the BP_ND_ estimates. Therefore, we conclude that *V*_T_ is a more reliable parameter for the quantification of [^11^C]GSK-189254 binding in the rat brain than BP_ND_.

Next, we evaluated the influence of constraining the *V*_B_ parameter in our analysis. Our findings suggest that fixing *V*_B_ at 0.05 could enhance the robustness of parameter estimates by reducing the SE and COV. Our data are in line with a PET study describing the kinetic analysis of [^11^C]GSK-189254 in the porcine brain.^39^ In this study, the 2T4k model and *V*_B_ as a parameter fixed to 5% were also found as the best model for fitting the data. Despite the fact that the regional blood flow and blood volume are known to vary in several brain regions of humans^50,52^ and animals^53^ and that in some regions, such as the cerebellum, the blood volume is smaller than 5%, reducing the number of fit parameters by the fixation of *V*_B_ does seem to improve the reliability of the fit. The good correlation of our selected model (2T4k-*V*_B_) with the cardinal compartment model (2T4k) suggests that the introduced bias as a result of fixing the volume of blood to 0.05 is small.

Interestingly, the [^11^C]GSK-189254 *V*_T_ values in the porcine and human brain were substantially higher than those observed in rats in our study, with *V*_T_ values ranging from 5 to 20,^39^ 26 to 119,^1^ and 1 to 5, respectively. These pronounced differences might originate from different H_3_ receptor expression levels or different affinities of [^11^C]GSK-189254 for the H_3_ receptor in the brains of different species and even different brain regions. In the study of the pig brain,^39^ the radioligand affinity (0.1 nM) and the density of the targeted receptor (H_3_) in the cerebellum, cortex, and striatum were reported, respectively, as 0.74, 2.05, and 2.65 nM. Moreover, the metabolic rate of the tracer may also vary between different species. Another explanation could be higher values for protein binding in rodents compared to that in humans (or pigs) resulting in lower values of *V*_T_.

Patlak graphical analysis was not able to describe the [^11^C]GSK-189254 data (data not shown), which is in accordance with the findings from compartmental modeling that indicated [^11^C]GSK-189254 is a reversible tracer in the brain of rats. In contrast, Logan graphical analysis was well able to fit the data. There was strong correlation between the *V*_T_ values estimated by Logan analysis with a t* of 30 min and the *V*_T_ values obtained with the optimal compartmental model. Although Logan graphical analysis takes advantage of the linearized equations and provides ease in computation compared to compartmental models, the mapping of fit parameters is a nonlinear estimation and would result in an underestimation of *V*_T_ values and increased noise.^54^ Both compartment models and Logan graphical analysis depend on blood sampling to generate a plasma input function. Since arterial blood sampling in rats is challenging and precludes the use of the technique in a longitudinal manner in imaging studies, we explored the use of simplified methods for data analysis that do not require an arterial input function.

Based on the lowest *V*_T_ and BP_ND_ values from compartmental modeling and the known distribution of H_3_ expression,^51^ the cerebellum and brainstem were identified as candidate (pseudo-)reference regions. These regions were evaluated to estimate outcome parameter(s) of [^11^C]GSK-189254 in the rat brain with a reference tissue-based model. The reference-tissue models SRTM and SRTM2, using the cerebellum and brainstem as the reference tissue, could fit the experimental data well, but the outcome parameters had a poor correlation with those derived from the compartment model. There might be three explanations for this lack of correlation: (1) these regions are not entirely devoid of H_3_ receptors which could be checked through immunostaining studies or blocking studies, (2) the *K*_1_/*k*_2_ ratios for the target region and the reference region are not identical, or (3) the BP_ND_ estimates from the 2T4k model are unreliable. In our study, the *K*_1_/*k*_2_ ratios in the reference regions were indeed significantly smaller than the *K*_1_/*k*_2_ ratios in target regions such as the striatum or frontal cortex (Supporting Information, Figure 2 and Supporting Information, Table 2). Apparently, at least one of the assumptions underlying the reference tissue models may have been violated for the two proposed reference regions, resulting in inaccurate estimates of BP_ND_. Despite this potential violation, BP_ND_ estimated with SRTM showed good to excellent correlations with the *V*_T_ and DVR estimated from the 2T4k-*V*_B_ model and also with the SUVr. In contrast, BP_ND_ derived from the 2T4k-*V*_B_ model was poorly correlated with all other measures of [^11^C]GSK-189254 binding [*V*_T_, DVR, BP_ND_(SRTM), SUV, and SUVr]. This suggests that the poor correlation between the BP_ND_ values derived from the 2T4k-*V*_B_ model and from SRTM is most likely due to the unreliable estimation of BP_ND_ (2T4k-*V*_B_) because of the small values estimated for *k*_3_ and *k*_4_, rather than the violation of the assumptions of the SRTM.

Perhaps, more reliable estimations of *k*_3_ and *k*_4_ could be made if the acquisition time was extended, but this requires a tracer with a longer half-life. The development of a ^18^F-labeled tracer for H_3_ receptors might indeed offer some advantages. Such a tracer would allow longer scanning times and transportation of the radioligand to nearby imaging centers. However, to the best of our knowledge, all ^18^F-labeled tracer candidates for H_3_ receptors failed with the exception of the inverse agonist developed by Hamill et al.^37^

An interesting finding of our study is the high correlation of the *V*_T_ of the selected compartment model with both the SUV_40–60_ and the SUVr with the cerebellum or brainstem as the reference region. One of the reasons that *V*_T_ slightly better correlates with SUVr than with SUV could be that some tracer delivery effects are compensated for when an SUVr is used. These findings can suggest that SUV_40–60_ and SUVr measurements might be acceptable parameters for the quantification of receptor binding of this tracer, when information from blood samples is not available, and thus could be considered as a simplified approach to the quantification of the regional binding of [^11^C]GSK-189254.

## Conclusions

This study investigated the binding kinetics of [^11^C]GSK-189254 to the histamine H_3_ receptor in the brain of rats with PET imaging. We demonstrated that the kinetics of [^11^C]GSK-189254 in the rat brain can be reliably described with *V*_T_ as determined with the 2T4k model considering the *V*_B_ parameter as a constant fixed to 0.05 or Logan graphical analysis with a t* of 30 min. BP_ND_ could not be reliably estimated from the 2T4k model, probably due to the unreliable estimation of the small *k*_3_ and *k*_4_ rate constants. Interestingly, *V*_T_ estimated from the 2T4k model showed a good correlation with SUV_40–60_ and the reference tissue-based parameters BP_ND_ calculated with SRTM or SRTM2, the DVR, and the SUVr with the cerebellum or brainstem as reference regions. These reference tissue approaches might therefore be acceptable alternatives in situations where arterial blood sampling is not possible.

## Supporting Information Available

Average and standard deviation of AIC values for the cardinal compartment models; efflux ratio of K_1_/k_2_ for the 2T4k and 2T4k-V_B_ compartment models; linear Pearson correlation of tissue-based reference regions of SRTM and SRTM2; one-way analysis of variance on estimated values for the K_1_/k_2_ ratio with Tukey post-hoc analysis; averaged SUV of [^11^C]GSK-189254, correlations between DVR, tissue-to-reference tissue ratio, and derived BP_ND_; and regression correlation analysis of the distribution volume (VT) and tissue-to-reference tissue ratio (PDF)
