# Supplementary material for: Pharmacokinetic Modeling of [11C]GSK-189254, PET Tracer Targeting H3 Receptors, in Rat Brain
Source: Mol Pharm. 2022 Feb 16;19(3):918–28. doi: 10.1021/acs.molpharmaceut.1c00889 (PMC8905578; doi:10.1021/acs.molpharmaceut.1c00889)
Supplement: Supplementary file 1 — mp1c00889_si_001.pdf [file mp1c00889_si_001.pdf]

Table 1: Average and standard deviation of AIC values for the cardinal compartment models 1T2k, 2T3k, and 2T4k

Brainregions	1T2K	2T3K	2T4K
Parietal-cortex	297.13±19.3	289.17±12.62	280.12±12.64
Temporal-cortex	305.75±18.39	296.05±16.64	278.72±27.06
Occipital-cortex	302.53±15.37	291.67±12.00	281.67±16.26
Frontal-cortex	296.56±18.92	287.48±13.12	275.62±13.53
Striatum	290.85±20.23	288.79±15.04	287.11±16.29
Amygdala	314.67±19.06	308.52±11.8	302.09±16.30
Cerebellum	319.97±12.97	285.44±9.50	267.72±17.13
Hippocampus	299.95±19.82	289.78±17.28	283.77±20.81
Hypothalamus	326.51±15.67	315.95±10.89	307.18±13.18
Brainstem	318.46±14.97	300.93±10.17	282.09±19.41
Midbrain	310.06±21.98	297.60±12.96	286.35±16.31
Thalamus	307.60±19.72	297.82±19.16	293.71±18.16
Whole-Brain	311.04±17.61	289.17±12.62	252.83±22.33

Table 2: The efflux ratio of K_1/k_2 for the 2T4k and 2T4k-V_B compartment models

Brainregions	K_1/k_2(2T4k)	K_1/k_2(2T4k-V_B)
Parietal-cortex	1.26±0.33	1.64±0.44
Temporal-cortex	1.13±0.3	1.12±0.24
Occipital-cortex	1.18±0.35	1.21±0.29
Frontal-cortex	1.29±0.66	1.39±0.46
Striatum	1.81±1.13	2.29±1.18
Amygdala	0.84±0.45	0.74±0.34
Cerebellum	0.90±0.11	0.87±0.10
Hippocampus	1.40±0.39	1.26±0.36
Hypothalamus	1.37±0.43	1.30±0.28
Brainstem	0.92±0.10	0.91±0.09
Midbrain	1.16±0.26	1.15±0.21
Thalamus	1.30±0.44	1.40±0.33
Whole-Brain	1.07±0.16	1.05±0.11

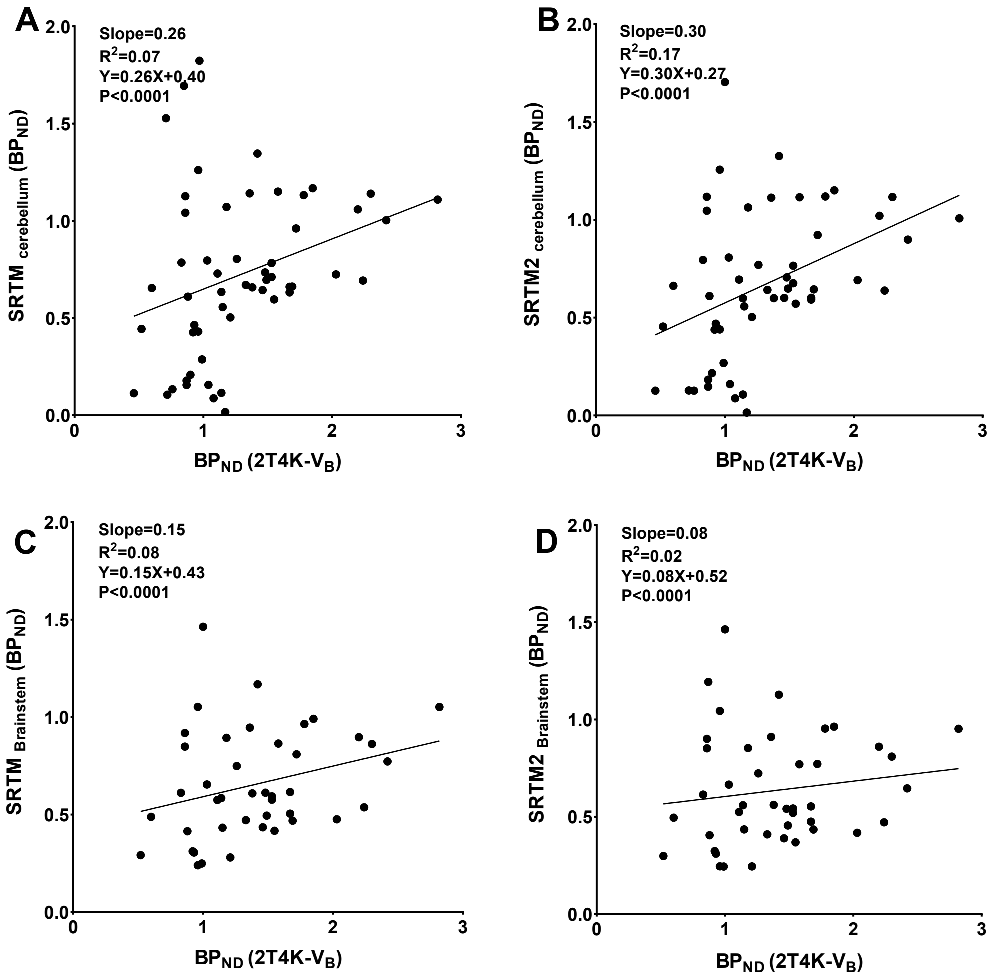

Figure 1. Linear Pearson correlation of tissue-based reference regions of SRTM (A and C) and SRTM2 (B and D) considering cerebellum (first row) and brainstem (second row) as reference regions with the optimal compartment model (2T4k-V_B).

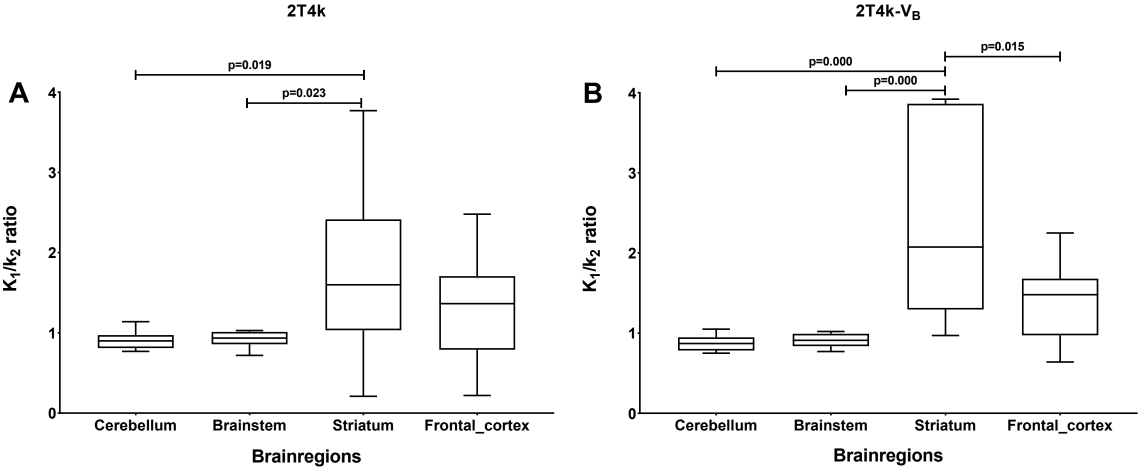

Figure 2. One way-ANOVA on estimated values for K_1/k_2 ratio with Tukey post-hoc analysis. The statistical analysis revealed that there were significant differences in K_1/k_2 ratio between the target regions (striatum, frontal cortex) and the reference regions (cerebellum, brainstem). Thus, the assumptions of equal K_1/k_2 ratio of the SRTM/SRTM2 model have been violated.

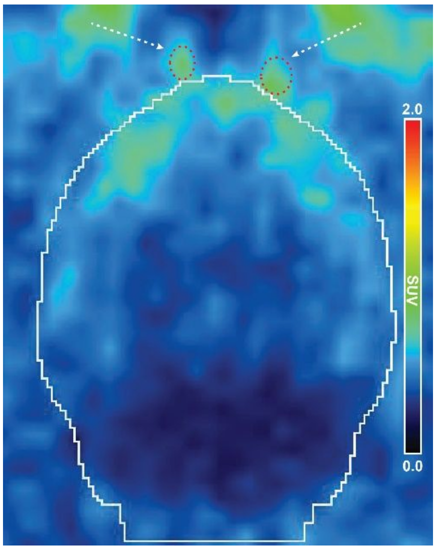

Figure 3. Averaged SUV of [^{11}C]GSK-189254 in a rat brain including the olfacutory bulbs and Harderian glands. The position of the two olfactory bulbs is indicated by dashed arrows.

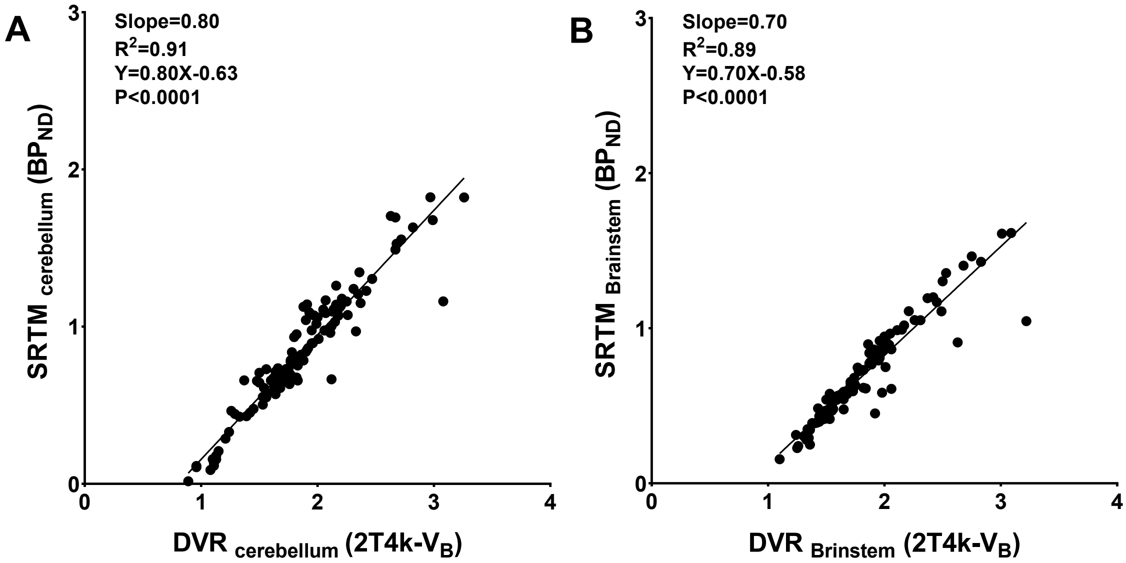

Figure 4. correlations between distribution volume ratio (DVR), tissue-to-reference tissue ratio, and derived binding potential (BP_{ND}) from reference tissue model considering either cerebellum or brainstem as (semi)-reference regions.

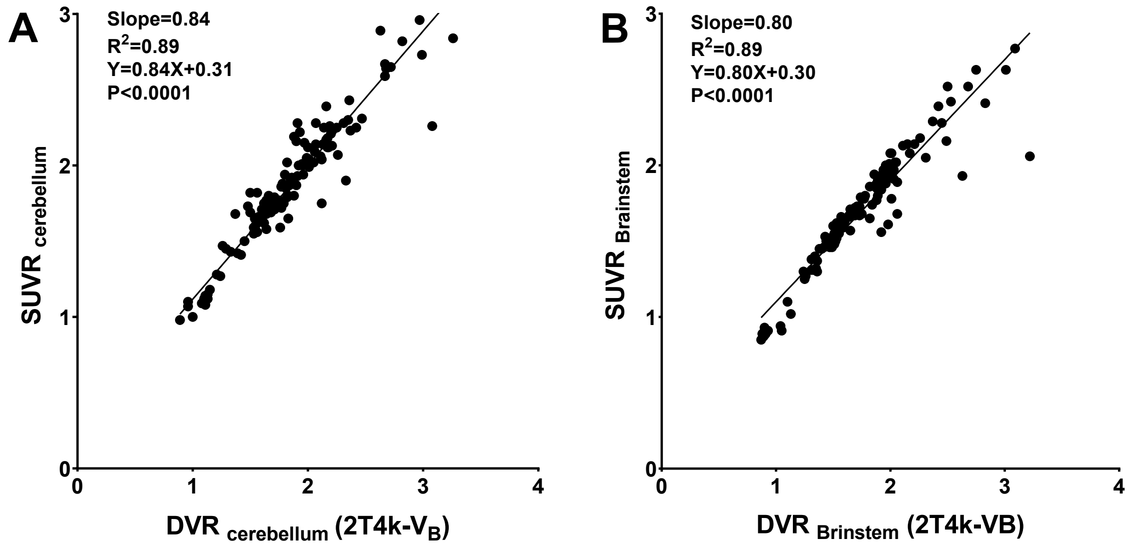

Figure 5. Regression correlation analysis of the distribution volume (V_T) and tissue-to-reference tissue ratio, either cerebellum or brainstem, of [^{11}C]GSK-189254 in individual regions of the rat brain
